# Supplementary material for: Modeling glioblastoma heterogeneity as a dynamic network of cell states
Source: Mol Syst Biol. 2021 Sep 16;17(9):e10105. doi: 10.15252/msb.202010105 (PMC8444284; doi:10.15252/msb.202010105)
Supplement: Supplementary file 6 — Source Data for Figure 5 [file MSB-17-e10105-s004.zip › Figure5A_sourcedata/GSEA_3071/nosigdb_stateIV.GseaPreranked.1622795164626/RICHARDS_NOWAKOWSKI_SCIENCE_2017_IN_CTX_CGE1_UPREG.html]

Details for gene set RICHARDS\_NOWAKOWSKI\_SCIENCE\_2017\_IN\_CTX\_CGE1\_UPREG[GSEA]

|  || Dataset | state53071 |
| Phenotype | NoPhenotypeAvailable |
| Upregulated in class | na\_pos |
| GeneSet | RICHARDS\_NOWAKOWSKI\_SCIENCE\_2017\_IN\_CTX\_CGE1\_UPREG |
| Enrichment Score (ES) | 0.46945566 |
| Normalized Enrichment Score (NES) | 2.831565 |
| Nominal p-value | 0.0 |
| FDR q-value | 0.0 |
| FWER p-Value | 0.0 |
Table: GSEA Results Summary

  

Fig 1: Enrichment plot: RICHARDS\_NOWAKOWSKI\_SCIENCE\_2017\_IN\_CTX\_CGE1\_UPREG      
 Profile of the Running ES Score & Positions of GeneSet Members on the Rank Ordered List

  

| PROBE | GENE SYMBOL | GENE\_TITLE | RANK IN GENE LIST | RANK METRIC SCORE | RUNNING ES | CORE ENRICHMENT || 1 | DCX |  |  | 4 | 2.752 | 0.0234 | Yes |
| 2 | DLX5 |  |  | 17 | 2.283 | 0.0412 | Yes |
| 3 | LINC00461 |  |  | 30 | 2.010 | 0.0565 | Yes |
| 4 | CHL1 |  |  | 37 | 1.968 | 0.0727 | Yes |
| 5 | DLX2 |  |  | 50 | 1.858 | 0.0867 | Yes |
| 6 | SOX11 |  |  | 56 | 1.811 | 0.1017 | Yes |
| 7 | DLX1 |  |  | 62 | 1.768 | 0.1162 | Yes |
| 8 | CDH2 |  |  | 68 | 1.735 | 0.1305 | Yes |
| 9 | DCLK2 |  |  | 74 | 1.704 | 0.1446 | Yes |
| 10 | DPYSL3 |  |  | 75 | 1.691 | 0.1594 | Yes |
| 11 | SDK2 |  |  | 85 | 1.618 | 0.1719 | Yes |
| 12 | TCF4 |  |  | 96 | 1.551 | 0.1836 | Yes |
| 13 | PROX1 |  |  | 126 | 1.468 | 0.1909 | Yes |
| 14 | CCDC88A |  |  | 135 | 1.429 | 0.2019 | Yes |
| 15 | RASSF2 |  |  | 140 | 1.400 | 0.2134 | Yes |
| 16 | TIAM2 |  |  | 141 | 1.393 | 0.2257 | Yes |
| 17 | CHD7 |  |  | 144 | 1.386 | 0.2375 | Yes |
| 18 | DLX6 |  |  | 145 | 1.384 | 0.2497 | Yes |
| 19 | ZEB2 |  |  | 164 | 1.339 | 0.2580 | Yes |
| 20 | PLCB1 |  |  | 168 | 1.323 | 0.2690 | Yes |
| 21 | NRXN3 |  |  | 170 | 1.321 | 0.2805 | Yes |
| 22 | SP9 |  |  | 189 | 1.281 | 0.2882 | Yes |
| 23 | GAD1 |  |  | 190 | 1.281 | 0.2995 | Yes |
| 24 | SLAIN1 |  |  | 203 | 1.257 | 0.3082 | Yes |
| 25 | GAS2L3 |  |  | 244 | 1.161 | 0.3106 | Yes |
| 26 | RBP1 |  |  | 254 | 1.136 | 0.3189 | Yes |
| 27 | GNAQ |  |  | 265 | 1.110 | 0.3267 | Yes |
| 28 | MEIS1 |  |  | 269 | 1.107 | 0.3359 | Yes |
| 29 | PFN2 |  |  | 270 | 1.107 | 0.3456 | Yes |
| 30 | PCDH9 |  |  | 301 | 1.034 | 0.3489 | Yes |
| 31 | MAGI1 |  |  | 355 | 0.969 | 0.3470 | Yes |
| 32 | RUFY3 |  |  | 376 | 0.929 | 0.3513 | Yes |
| 33 | SOX4 |  |  | 377 | 0.928 | 0.3595 | Yes |
| 34 | TOP2B |  |  | 382 | 0.924 | 0.3668 | Yes |
| 35 | PDZRN3 |  |  | 405 | 0.904 | 0.3705 | Yes |
| 36 | ARX |  |  | 415 | 0.891 | 0.3766 | Yes |
| 37 | ELOVL6 |  |  | 437 | 0.867 | 0.3801 | Yes |
| 38 | TCEAL2 |  |  | 464 | 0.845 | 0.3825 | Yes |
| 39 | RTN1 |  |  | 471 | 0.836 | 0.3887 | Yes |
| 40 | ZNF704 |  |  | 498 | 0.818 | 0.3908 | Yes |
| 41 | ZKSCAN1 |  |  | 524 | 0.794 | 0.3929 | Yes |
| 42 | MYL6B |  |  | 529 | 0.786 | 0.3991 | Yes |
| 43 | RCN2 |  |  | 533 | 0.782 | 0.4054 | Yes |
| 44 | BLOC1S1 |  |  | 570 | 0.750 | 0.4049 | Yes |
| 45 | PTOV1 |  |  | 580 | 0.740 | 0.4097 | Yes |
| 46 | GABARAPL2 |  |  | 583 | 0.739 | 0.4158 | Yes |
| 47 | TMOD2 |  |  | 591 | 0.732 | 0.4209 | Yes |
| 48 | GOLGA7 |  |  | 619 | 0.714 | 0.4219 | Yes |
| 49 | LEPROTL1 |  |  | 624 | 0.712 | 0.4274 | Yes |
| 50 | SLC39A6 |  |  | 629 | 0.708 | 0.4328 | Yes |
| 51 | JAKMIP2 |  |  | 657 | 0.685 | 0.4336 | Yes |
| 52 | ARID1B |  |  | 686 | 0.667 | 0.4340 | Yes |
| 53 | AGO2 |  |  | 692 | 0.663 | 0.4389 | Yes |
| 54 | RBM5 |  |  | 697 | 0.662 | 0.4439 | Yes |
| 55 | TNRC6B |  |  | 698 | 0.662 | 0.4497 | Yes |
| 56 | ATF7IP |  |  | 711 | 0.653 | 0.4532 | Yes |
| 57 | POU2F1 |  |  | 718 | 0.649 | 0.4577 | Yes |
| 58 | DHX36 |  |  | 733 | 0.641 | 0.4606 | Yes |
| 59 | PPP1R9A |  |  | 768 | 0.621 | 0.4594 | Yes |
| 60 | CTNND2 |  |  | 775 | 0.618 | 0.4637 | Yes |
| 61 | MIDN |  |  | 795 | 0.604 | 0.4653 | Yes |
| 62 | CASD1 |  |  | 802 | 0.602 | 0.4695 | Yes |
| 63 | STMN4 |  |  | 876 | 0.570 | 0.4602 | No |
| 64 | SLC6A1 |  |  | 927 | 0.549 | 0.4553 | No |
| 65 | SRGAP2C |  |  | 981 | 0.530 | 0.4496 | No |
| 66 | CRBN |  |  | 1018 | 0.515 | 0.4472 | No |
| 67 | SETD2 |  |  | 1021 | 0.514 | 0.4513 | No |
| 68 | APLP1 |  |  | 1035 | 0.510 | 0.4532 | No |
| 69 | UQCR11 |  |  | 1114 | 0.488 | 0.4423 | No |
| 70 | MLF2 |  |  | 1118 | 0.487 | 0.4460 | No |
| 71 | BTG1 |  |  | 1123 | 0.486 | 0.4495 | No |
| 72 | METTL9 |  |  | 1177 | 0.472 | 0.4433 | No |
| 73 | PRKCA |  |  | 1214 | 0.461 | 0.4404 | No |
| 74 | GOLGA4 |  |  | 1231 | 0.456 | 0.4413 | No |
| 75 | DCAF7 |  |  | 1251 | 0.452 | 0.4415 | No |
| 76 | NDRG4 |  |  | 1266 | 0.449 | 0.4428 | No |
| 77 | PAXBP1 |  |  | 1303 | 0.440 | 0.4396 | No |
| 78 | C5orf24 |  |  | 1315 | 0.437 | 0.4413 | No |
| 79 | MAGEH1 |  |  | 1337 | 0.432 | 0.4410 | No |
| 80 | SHPRH |  |  | 1339 | 0.430 | 0.4446 | No |
| 81 | TNRC6A |  |  | 1344 | 0.429 | 0.4476 | No |
| 82 | RSRC1 |  |  | 1347 | 0.428 | 0.4510 | No |
| 83 | PHF20L1 |  |  | 1360 | 0.426 | 0.4524 | No |
| 84 | SFXN1 |  |  | 1374 | 0.422 | 0.4536 | No |
| 85 | SERINC5 |  |  | 1383 | 0.421 | 0.4557 | No |
| 86 | MOB1B |  |  | 1425 | 0.410 | 0.4513 | No |
| 87 | MEF2A |  |  | 1547 | 0.379 | 0.4311 | No |
| 88 | MOB3B |  |  | 1552 | 0.377 | 0.4336 | No |
| 89 | SNRPN |  |  | 1634 | 0.362 | 0.4210 | No |
| 90 | MGA |  |  | 1659 | 0.357 | 0.4195 | No |
| 91 | MTSS1 |  |  | 1708 | 0.348 | 0.4132 | No |
| 92 | TCF3 |  |  | 1709 | 0.348 | 0.4162 | No |
| 93 | TCEAL8 |  |  | 1712 | 0.348 | 0.4189 | No |
| 94 | SMDT1 |  |  | 1718 | 0.347 | 0.4210 | No |
| 95 | WDR83OS |  |  | 1761 | 0.337 | 0.4157 | No |
| 96 | SPEN |  |  | 1793 | 0.332 | 0.4126 | No |
| 97 | YLPM1 |  |  | 1824 | 0.327 | 0.4097 | No |
| 98 | ERBB4 |  |  | 1843 | 0.325 | 0.4090 | No |
| 99 | DCLK1 |  |  | 1860 | 0.321 | 0.4087 | No |
| 100 | YEATS2 |  |  | 1885 | 0.316 | 0.4068 | No |
| 101 | MLLT11 |  |  | 1932 | 0.308 | 0.4005 | No |
| 102 | LRCH2 |  |  | 1962 | 0.304 | 0.3976 | No |
| 103 | USP34 |  |  | 2027 | 0.292 | 0.3877 | No |
| 104 | SMARCA1 |  |  | 2078 | 0.286 | 0.3804 | No |
| 105 | PUM2 |  |  | 2099 | 0.284 | 0.3790 | No |
| 106 | PDE4D |  |  | 2100 | 0.283 | 0.3815 | No |
| 107 | NECAP1 |  |  | 2127 | 0.280 | 0.3789 | No |
| 108 | DHPS |  |  | 2144 | 0.277 | 0.3782 | No |
| 109 | C2orf68 |  |  | 2213 | 0.266 | 0.3673 | No |
| 110 | CNOT2 |  |  | 2256 | 0.261 | 0.3614 | No |
| 111 | ZNF37A |  |  | 2301 | 0.255 | 0.3551 | No |
| 112 | COX6B1 |  |  | 2309 | 0.253 | 0.3560 | No |
| 113 | SPIN1 |  |  | 2358 | -0.254 | 0.3488 | No |
| 114 | ERH |  |  | 2555 | -0.276 | 0.3130 | No |
| 115 | EIF1AX |  |  | 2584 | -0.280 | 0.3100 | No |
| 116 | RIF1 |  |  | 2770 | -0.302 | 0.2766 | No |
| 117 | PLRG1 |  |  | 2872 | -0.315 | 0.2597 | No |
| 118 | FRY |  |  | 2899 | -0.319 | 0.2574 | No |
| 119 | TFAM |  |  | 2902 | -0.319 | 0.2599 | No |
| 120 | RBX1 |  |  | 2951 | -0.325 | 0.2534 | No |
| 121 | NUP50 |  |  | 2954 | -0.326 | 0.2558 | No |
| 122 | PHTF2 |  |  | 2993 | -0.332 | 0.2513 | No |
| 123 | TBPL1 |  |  | 3004 | -0.333 | 0.2523 | No |
| 124 | ZC3H15 |  |  | 3093 | -0.344 | 0.2382 | No |
| 125 | TIMM44 |  |  | 3128 | -0.350 | 0.2347 | No |
| 126 | PSMC2 |  |  | 3152 | -0.353 | 0.2333 | No |
| 127 | IVNS1ABP |  |  | 3154 | -0.353 | 0.2362 | No |
| 128 | TXNL1 |  |  | 3177 | -0.356 | 0.2350 | No |
| 129 | POLR1D |  |  | 3188 | -0.358 | 0.2362 | No |
| 130 | NSA2 |  |  | 3191 | -0.359 | 0.2390 | No |
| 131 | PSMD14 |  |  | 3196 | -0.359 | 0.2414 | No |
| 132 | KITLG |  |  | 3197 | -0.360 | 0.2445 | No |
| 133 | UBE2L3 |  |  | 3222 | -0.363 | 0.2431 | No |
| 134 | RPL36AL |  |  | 3275 | -0.371 | 0.2362 | No |
| 135 | CDC37 |  |  | 3286 | -0.374 | 0.2375 | No |
| 136 | SCNM1 |  |  | 3370 | -0.386 | 0.2247 | No |
| 137 | NBPF9 |  |  | 3426 | -0.395 | 0.2175 | No |
| 138 | ATP6V1D |  |  | 3441 | -0.396 | 0.2182 | No |
| 139 | ARFGAP3 |  |  | 3537 | -0.416 | 0.2034 | No |
| 140 | HIPK1 |  |  | 3553 | -0.419 | 0.2041 | No |
| 141 | DDX27 |  |  | 3573 | -0.423 | 0.2042 | No |
| 142 | EXOSC10 |  |  | 3738 | -0.452 | 0.1762 | No |
| 143 | MDH2 |  |  | 3798 | -0.466 | 0.1688 | No |
| 144 | UBE2A |  |  | 3934 | -0.494 | 0.1468 | No |
| 145 | CSTB |  |  | 3946 | -0.497 | 0.1490 | No |
| 146 | RRP15 |  |  | 4034 | -0.521 | 0.1366 | No |
| 147 | MYL12B |  |  | 4093 | -0.539 | 0.1301 | No |
| 148 | CACYBP |  |  | 4109 | -0.542 | 0.1319 | No |
| 149 | PAK1IP1 |  |  | 4126 | -0.546 | 0.1336 | No |
| 150 | NCL |  |  | 4171 | -0.560 | 0.1299 | No |
| 151 | RPP30 |  |  | 4262 | -0.586 | 0.1175 | No |
| 152 | CITED2 |  |  | 4303 | -0.598 | 0.1150 | No |
| 153 | SF3A3 |  |  | 4327 | -0.605 | 0.1158 | No |
| 154 | POLE3 |  |  | 4336 | -0.610 | 0.1196 | No |
| 155 | RBM17 |  |  | 4362 | -0.622 | 0.1202 | No |
| 156 | RND3 |  |  | 4402 | -0.635 | 0.1182 | No |
| 157 | EIF3J |  |  | 4426 | -0.643 | 0.1194 | No |
| 158 | TMSB10 |  |  | 4451 | -0.654 | 0.1205 | No |
| 159 | PDE4DIP |  |  | 4715 | -0.804 | 0.0763 | No |
| 160 | NR2F2 |  |  | 4870 | -0.937 | 0.0545 | No |
| 161 | NGRN |  |  | 4879 | -0.944 | 0.0612 | No |
| 162 | NF2 |  |  | 4930 | -0.995 | 0.0603 | No |
| 163 | DDX10 |  |  | 5008 | -1.109 | 0.0550 | No |
Table: GSEA details [plain text format]

  

Fig 2: RICHARDS\_NOWAKOWSKI\_SCIENCE\_2017\_IN\_CTX\_CGE1\_UPREG: Random ES distribution      
 Gene set null distribution of ES for **RICHARDS\_NOWAKOWSKI\_SCIENCE\_2017\_IN\_CTX\_CGE1\_UPREG**
